# Yield, profitability, and prospects of irrigated Boro rice cultivation in the North-West region of Bangladesh

**DOI:** 10.1371/journal.pone.0250897

**Published:** 2021-04-29

**Authors:** Mohammed Mainuddin, Md. Mahbubul Alam, Md. Maniruzzaman, Md. Jahangir Kabir, Mohammad A. Mojid, Md. Masud Hasan, Erik J. Schmidt, Md. Towfiqul Islam

**Affiliations:** 1 CSIRO Land and Water, Black Mountain, Canberra, Australian Capital Territory, Australia; 2 Bangladesh Rice Research Institute, Gazipur, Bangladesh; 3 Bangladesh Agricultural University, Mymensingh, Bangladesh; 4 Australian National University, Canberra, Australia; 5 University of Southern Queensland, Toowoomba, Queensland, Australia; Soil and Water Resources Institute ELGO-DIMITRA, GREECE

## Abstract

The North-West (NW) region of Bangladesh is pivotal for the country’s agricultural development, mainly in producing irrigated Boro rice. However, increasing cost of irrigation water, fertilizers, labour and other inputs, and the spatio-temporal variation in actual yield, market price and profitability of rice, have added uncertainty to the sustainability of Boro rice cultivation. In this study, we evaluated the productivity, profitability, and prospect of Boro rice production using comprehensive field data collected directly from 420 farmers’ fields over two consecutive seasons (2015–16 and 2016–17), across seven geographically distributed locations in the NW region. We also analyzed the risk and return trade of popular Boro rice cultivars using Monte-Carlo simulation. The results show that there were significant (p≤0.05) variations in rice yield between sites, irrigation pump-types, and rice varieties, with Hybrid rice and BRRI dhan29 producing highest yields (6.0–7.5 t/ha). Due to different pricing systems, the cost of irrigation water varied from site to site and from year to year, but always comprised the highest input cost (20–25% of total production). The total paid-out cost, gross benefit, and gross income of rice significantly (p≤0.05) differed between sites, type of irrigation pumps, rice varieties, transplanting dates, and two cropping years. The variations in observed yield and profitability reveal considerable scope to improve rice production systems. Market variation in the price of rice affected overall profitability significantly. Probability and risk analysis results show that Minikit and BRRI dhan29 are the most stable varieties for yield and profitability. Hybrid rice, which has the maximum attainable yield among the cultivated rice varieties, also has the risk of negative net income. Based on the analysis, we discussed ways to improve yield and profitability and the prospect of Boro rice cultivation in the region. The study provides valuable information for policy-makers to sustain irrigated rice cultivation in both the NW region and nationally.

## Introduction

Rice is the predominant crop in the Asia-Pacific region. The region produces and consumes over 90 percent of the world’s rice [1, 2]. Rice cultivation is the dependable source for employment and income for millions of households [3]. However, in recent years, overall profitability has been reducing due to rising input prices and the increasing cost of labour discouraging rice production [4]. Reducing income volatility and increasing profitability is an important step to increase social welfare and sustainability of rice production [5]. Yield is intricately linked with profitability, and yield gains have helped keep rice cultivation profitable, especially after 2005 in Bangladesh [6].

Bangladesh has achieved major advances in agricultural development over the last 30 years, especially in rice production. Bangladesh is self-sufficient in rice [7, 8], which is the staple food, with an average per capita consumption of 134 kg per annum, compared to the world average of 57 kg per annum [9]. It is the most dominant crop and generates a major share of farmers’ income and employment [10, 11]. It is the predominant crop in the three main crop-growing seasons in Bangladesh; Aus rice is grown during March to June, Aman rice during June/July to October/November and irrigated Boro rice during January to April/May (the Rabi season popularly known as Boro season). Both Aus and Aman rice are mainly rainfed or only occasionally irrigated. The Rabi season has very little rainfall and hence the Boro rice is fully irrigated. The average yield of rice has increased linearly over the past four decades [7]. Total production of rice has increased from 11.6 million tons in 1977 to 34 million tons (almost 3 fold) in 2016 while the population during this period increased from 76 million to 168 million (2.2 fold) [12, 13]. Rice production has intensified through the introduction of high-yielding and hybrid Boro rice varieties, cultivated in the dry season using irrigation, as well as the increased application of fertilizer, pesticides, herbicides, and better crop management. Currently, 61% of the total cropped area in the Rabi season is under Boro cultivation which contributes 55% to total rice production [12]. Extensive Boro rice cultivation has been possible due to expansion of groundwater irrigation by shallow tubewells (STWs) and deep tubewells (DTWs). The increase in rice production has outpaced the increase in population, making agriculture a major contributor to poverty alleviation in Bangladesh since 2000 [14].

The North-West (NW) region of Bangladesh produces over 1/3 of the of the country’s total rice despite covering only 23.5% of the country’s total area [12]. It has 30% of the net cultivable area (NCA) and 40% of the total irrigated area of the country [15]. The region also has the highest average yield of rice [16]. The average cropping intensity is also higher in this region (205%) than the country-average value (194%) [17]. The population of Bangladesh has been projected to increase to 202 million in 2050 from the current 168 million [8, 18]. To meet the growing demand for food, increase in yield of Boro rice is required [7]. The positive impacts of irrigation on intensification of agriculture, crop productivity and farm income have been well-documented for this region and also for other regions of Bangladesh [e.g., 7, 19–21]. Consequently, sustaining the current growth in irrigated rice production in NW Bangladesh is a national priority of the government of Bangladesh [22]. Increasing food production from the shrinking resources base, specifically water and land resources, to keep pace with the growing population, will be a key challenge for the country in future [7, 8, 19, 21, 23].

Reducing variation in rice productivity or yield in the farmers’ fields offers valuable opportunities to produce additional rice, even using available technologies [2]. Yield gap or variation is the difference between the farm-level actual yield and the maximum yield attainable at experimental fields with no physical, biological, or economic constraints and with the best-known management practices for a given time and in a given environment [24, 25]. In most cases, the farm-level rice yield remains below the attainable yield due to poor agronomic management [25, 26]. The influence of agronomic factors is related to complex economic, social, and political processes [25]. This is an important aspect when considering rice production for the semi-subsistence farming systems in Bangladesh [27]. To address this, variation in actual yield between individual farmers, sites, irrigation pump-types, and transplanting dates for the same rice variety need to be considered.

While making decisions for rice cultivation, farmers always consider the expected returns against the cost of production [28] (Dillon and Hardaker, 1993). However, due to fluctuation in the market price of rice [6, 29], volatility in rice income has become the norm among rice farmers in Bangladesh [5]. For effective policy interventions, it is important to analyze the cost, actual yield, profitability, and risk of rice cultivation under varying farming conditions (e.g., locations, different varieties, input applications, irrigation water source, planting dates, etc.). Many studies can be found dealing with similar issues of rice cultivation, such as yield or productivity and sustainability in rice farming [e.g. 23, 30–40], factors influencing productivity [e.g. 8, 10, 34], farm household income and expenditure [e.g. 4, 5, 41] and their relations with poverty and livelihoods [e.g. 6, 42–44]. These studies are either based on the combination of secondary data available in the Statistical Yearbooks [e.g. 12, 17], Household Income and Expenditure Surveys [e.g. 45], published every year by the Bangladesh Bureau of Statistics, and household surveys through a questionnaire [e.g. 5, 10, 34, 40], or farm level field experiments [e.g. 30, 31, 41]. None of these studies uses actual field data informing the factors affecting yield, profitability, and risk and none deal comprehensively with both yield and economic viability, and risk of cultivating different varieties of rice in Bangladesh. No field-based comprehensive study has yet dealt with farmers’ practice and the profitability of rice crops in Bangladesh [27]. This study provides a detailed determination of the state (productivity, profitability, and prospects or risk) of irrigated Boro rice cultivation, based on intensive field observations conducted during two consecutive seasons (2015–16, and 2016–17) in 420 fields (235 in 2015–16 and 185 in 2016–17) across 7 locations in the NW region of Bangladesh. The specific objectives were: (i) to assess the variability in the actual yield of rice at different sites for different rice varieties, irrigation well operating modes and transplanting dates, (ii) to identify the cause(s) of productivity variations, (iii) to estimate the profitability and risk of rice cultivation, and (iv) to make suggestions for improving productivity and profitability of irrigated Boro rice cultivation.

## Methodology

### Location

**Fig 1:**
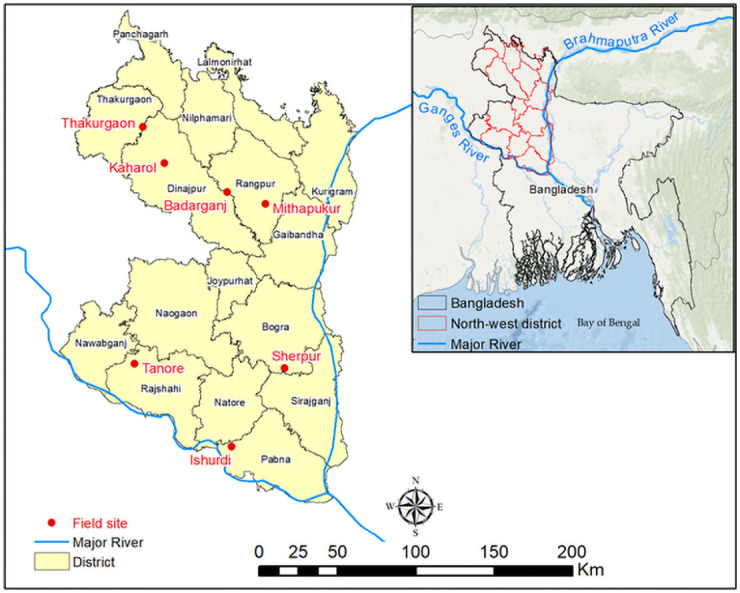
Location of the selected sites (solid circles) for monitoring and data recording.

The NW region of Bangladesh comprising 23.5% (34,515 km^2^) of the country’s total area is divided into 16 administrative districts (Fig 1). The annual rainfall in the region varies from 1,273 to 2,515 mm (1985–2010). The Rajshahi area has the lowest (1,428 mm) and Rangpur area has the highest (2,262 mm) average annual rainfall. Most of the rainfall (82%) occurs during monsoon (May–October) and the rest (18%) occurs during the dry season (November–April). The monthly average temperature ranges from 25°C to 35°C in the hottest period and from 9°C to 15°C in the coolest period [46]. Annual average relative humidity is 78% with the lowest monthly average (62%) in March and the highest (87%) in July [46]. The average reference evapotranspiration (ET_o_) in the region is 1,309 mm. which is close to the country’s highest ET_o_ (1,334 mm) observed in the South-West region. ET_o_ varies both spatially and temporally (CV = 6–8%) with the highest average value of 1,366 mm in Ishurdi and the lowest average value of 1,251 mm in Rangpur.

### Site selection

Several criteria were adopted in selecting the sites, including (i) a good geographical spread within NW Bangladesh, (ii) a combination of DTWs and/or STWs covering at least 10–20 ha agricultural lands bounded by village homes, (iii) both diesel- and electrically-operated STWs, and DTWs were considered (iv) irrigation is done predominantly for Boro rice, (v) a range of water pricing systems, and (vi) the sites are convenient for communication. Four water pricing systems were considered. These were share of crop as water charge, usage of a smart card, land area-based fixed water charge, and diesel paid by farmer plus a land area-based fixed charge. Rajshahi, Pabna, Bogra, Rangpur, Dinajpur and Thakurgaon districts were selected in the 1^st^ stage of monitoring and data collection (2015–16). DTW is the predominant irrigation technology in Rajshahi and Thakurgaon sites. STW irrigation is predominant in the other four sites, although both DTWs and STWs are used. Most of the farmers in these sites are marginal and tenant farmers and grows Boro rice in the dry season. Most of the farmers buy water from the pump owner to irrigate their crops. There is a vibrant water market.

A total of 14 STWs and 3 DTWs were selected in the six sites (Fig 1). All these sites were selected following the ethical guidelines of implementing organizations, CSIRO and Bangladesh Rice Research Institute (BRRI). BRRI conducted the study at the field level, which is a government research organization mandated to carry out rice related research in the farmers’ field. The ethics permission was given by CSIRO and BRRI. The study was carried out on farmers’ fields and the owner of the land gave permission to conduct the study on these sites. Field studies did not involve endangered or protected species. Monitoring and data recording started at the beginning of the crop season (November in 2015). STWs have small command area so all the fields irrigated by a STW were considered for investigation. The command areas of the DTWs are quite large and water is supplied through buried pipes. There are outlets at certain intervals through which water is delivered to the farm channels for the farmers to irrigate their fields. So, the command areas of three outlets (at the head, middle and tail end) in each DTW site were considered for investigation. The details of the STWs and DTWs and their command areas are provided in Table 1. Considering the large volume of data collection, the monitoring was not continued in 2016–17 in Tanore and Kaharol and the number of STWs in Mithapukur and Ishurdi was reduced. However, one additional site, where irrigation pumps are operated by solar power, was selected at Badarganj Upazilla of Rangpur district (Table 1) for data collection in 2016–2017. A total of 235 fields covering 35.06 ha and 185 fields covering 24.62 ha were monitored respectively in 2015–16 and 2016–17. So, our study is based on 425 farmers over two seasons. Several similar studies are conducted based on the data of one year. For example, Shah et al. [47] considered 425 farmers in a year of which 79 were non-adopters and 346 were adopters of hybrid rice. However, in the analysis they did not consider non-adopters. Rahman and Parkinson [36] used 380 samples from 3 study locations for one crop year (1996). A total number of 390 households from 15 villages were surveyed in a season by Roy et al. [38] to assess the rice farming sustainability in Bangladesh. All these evidences justify the adequacy of the data for this study.

**Table 1:** Summary information of the tubewells in the selected sites during Boro rice growing season (November to May) of 2015–16 and 2016–17.

Site	Types of tubewell	Power sources	Water pricing method	Discharge capacity (L/s)	2015–16		2016–17	
					No. of plots	Total area (ha)	No. of plots	Total area (ha)
Thakurgaon	DTW-1	Electric	Taka 110/hour	58.0	18	3.85	18	2.54
Kaharol	STW-1	Electric	Fixed rate, Taka 14,280 /ha	13.2	8	2.02		
	STW-2	Diesel	Fixed rate, Taka 17,290/ha	8.9	5	0.47		
	STW-3	Electric	Fixed rate, Taka 14,280 /ha	14.0	11	1.80		
Mithapukur	STW-1	Electric	Fixed rate, Taka 9880/ha	14.8	30	2.66	35	3.57
	STW-2	Diesel	Pump charge Taka 5,930 to 7,900/ha, farmers bring their own fuel	8.8	13	1.17	13	1.04
	STW-3	Diesel		9.4	10	1.17	14	1.57
	STW-4	Diesel		9.97	16	2.21		
Sherpur	STW-1	Diesel	Fixed rate, Taka 22,230/ha	7.1	10	1.28	19	2.16
	STW-2	Diesel		7.2	13	1.84	8	0.88
	STW-3	Diesel		7.3	29	3.04	19	2.97
Ishurdi	STW-1	Electric	Crop share, pump owner takes 25% of the crop	12.9	6	1.87	19	3.97
	STW-2	Diesel		14.0	19	2.94		
	STW-3	Electric		15.0	25	4.60	20	3.45
Tanore	DTW-1	Electric	120–140 Taka/hour	17.0	10	2.46		
	DTW-2	Electric		20.0	12	1.68		
Badarganj	STW	Solar	Fixed rate, Taka 17,290/ha	9.0			20	2.47
**Total**					**235**	**35.06**	**185**	**24.62**

### Data monitoring

We appointed a person to work full time at each site for the total project period to record the data. We used a pretested structured form to record inputs and labour used for intercultural operations (seed-bed preparation to winnowing and storing) of Boro rice in each field under farmers’ management. For example, fertilizer is used in 1 to 3 doses (during land preparation, 1^st^ top dressing, 2^nd^ top dressing) depending on the type of fertilizer. We recoded the price paid by the farmer to purchase fertilizers (urea, TSP, Potassium, Zinc, etc.) used in each application and the labour required to apply the fertilizer to the field. The total fertilizer cost was then calculated by summing up the total cost of purchasing fertilizer and the labour cost. Also, we recorded data on land ownership, land type, land area, soil type, cropping patterns, year of cultivation, crop types and varieties, date of transplanting and harvesting, type of irrigation pump, and quantity, actual yield, and price of rice.

#### Cultivated rice varieties

Among the seven study sites, the maximum number of rice fields (69 fields in 2015–16 and 62 fields in 2016–17) was in Mithapukur and minimum number (18 fields) was in Thakurgaon (Table 2). The average field size was higher (0.18 to 0.21 ha) in Ishurdi, Kahrol, Tanore and Thakurgaon than in the other sites. Mithapukur had the lowest average field size (0.10 ha), which was similar to that in Sherpur (0.12 ha). In the sites, farmers cultivated six high yielding rice varieties, BRRI dhan28, BRRI dhan29, Hybrid (3 varieties of hybrid rice such as Hera, Tej Gold and BRRI Hybrid dhan2) rice, Minikit, Jirashail, and Kajallata, in 2015–16 (Table 2). Farmers cultivated Jirashail only in Tanore and Kajallata in Sherpur in 2015–16. The growth duration (from sowing seed to the nursery to harvest) of BRRI dhan29 and Hybrid was 160–165 days and the growth duration of other varieties was around 140 days.

**Table 2:** Number of fields with different Boro rice varieties at the seven selected sites.

Variety	Ishurdi		Sherpur		Mithapukur		Thakurgaon		Tanore	Kaharol	Badarganj
	2015–16	2016–17	2015–16	2016–17	2015–16	2016–17	2015–16	2016–17	2015–16	2015–16	2016–17
BRRI dhan28	–	–	–	1	28	32	5	–	–	24	15
BRRI dhan29	44	18	–	–	4	4	13	18	–	–	3
Hybrid	6	–	–	–	37	26	–	–	–	–	2
Jirashail	–	–	–	–	–	–	–	–	22	–	–
Kajallata	–	–	21	–	–	–	–	–	–	–	–
Minikit^1^	–	21	31	45	–	–	–	–	–	–	–
Total	50	39	52	46	69	62	18	18	22	24	20

Note:

^1^ There was a program under which farmers were given a package or kit which included some seeds of an unknown rice variety from India. That variety got popular with the farmer which is popularly known as Minikit.

Farmers cultivated a maximum of two rice varieties in each site except in Mithapukur where a third variety was grown in four of the monitored fields and in Badarganj where a third rice variety was grown in two of the monitored fields. BRRI dhan28 and BRRI dhan29 were cultivated in half (50.2%) of the total fields in 2015–16. Minikit was cultivated in 13.2%, Kajallata in 8.9% and Jirashail in 9.4% of the fields. Hybrid rice varieties are now commercially available and becoming popular among the farmers due to their higher attainable yield. The Hybrid rice covered 18.3% of the total fields, mainly in Mithapukur site in 2015–16. In 2016–17, BRRI dhan28 and BRRI dhan29 were cultivated in 86 (46%) fields, mostly in Ishurdi, Mithapukur, Thakurgaon and Badarganj sites. The observed spatial distribution of the rice varieties indicates dominancy of certain rice varieties in certain sites as shown in Table 2.

#### Rice transplanting time

In 2015–16, transplanting of Boro rice started early in the season in late December and was completed by the end of February. with 80.5% of fields transplanted from 16 January to 15 February. During 2016–17, 93% of fields were transplanted during mid-January to mid-February. Transplanting time was also dependent on location and variety. For the analysis, we divided the transplanting period into 3 classes; January (including few fields transplanted in late December), 1–14 February and 15–28 February.

### Enterprise budgeting and probability of exceedance

Input use patterns were recorded for each intercultural operation of Boro rice production as described earlier, from seedling development in the nursery to temporary initial storage of produce after harvesting. Total paid-out cost of rice production is the sum of nine input cost items, which are seed, nursery preparation, land preparation (tillage), transplanting (including seedling uprooting), fertilizer (total of purchase and application), herbicide (including weeding), pesticide, irrigation, harvesting (cutting, carrying and threshing), and winnowing (include drying). The total gross benefit of rice was calculated as the sum of the market value of grain and straw yields at farm gate price at harvest time. Enterprise budgets for Boro rice were developed using [48] approach, which distinguishes between ‘paid-out cost’ and ‘unpaid cost’ based on purchased inputs and for family supplied inputs (i.e. imputed cost), respectively. Herdt [48] used enterprise return as ‘gross income’ and ‘net income’. Gross income is the gross benefit minus paid-out cost and ‘net income’ is the gross income minus imputed cost. Analysis of Variance (ANOVA) was used to compare the mean total paid-out cost of production, gross benefit, gross income, productivity and profitability of Boro rice across the sites, pump-types (electricity and diesel operated STWs and DTWs), rice cultivars and transplanting dates at 5% probability level (p≤0.05).

The exceedence probability of actual yield and gross income was calculated using the following equation (https://sciencing.com/calculate-exceedance-probability-5365868.html):

$$Exceedance\;probability,\;P(\%)=100\times \frac{m}{n+1}$$ (1)

Where, m is the rank of actual yield or gross income of a particular field, n is the total number of fields.

### Stochastic budgeting for risk accounting

To evaluate the riskiness of major Boro cultivars such as BRRI dhan28, BRRI dhan29, Hybrid rice and Minikit, stochastic budgets were constructed. The software @RISK was used to derive cumulative density functions (CDFs) of gross income and net income. @RISK analyze risk using Monte Carlo simulation and shows all possible outcomes for any situation and how likely they are to occur (https://www.palisade.com/risk/default.asp). That helps to judge which risk to take on and which ones to avoid (https://www.palisade.com/risk/default.asp). Monte Carlo simulation [49] was run following observed yearly variation in yield and price for two consecutive cropping seasons (2015–16 and 2016–17). Poor yield was defined as the average yield of the lowest 20% of the monitored fields’ yield, medium yield was the average of the middle 60%, and the high yield was the mean of the best 20%. Similarly, the low, average, and high prices of the cultivars for the stochastic budgets were taken. For simulating each CDF, 10,000 (maximum possible) iterations were used since it increases stability of the distribution [50]. Simple stochastic dominance rules [28, 51] of comparing the CDFs of the alternative cropping options were applied for risk analysis.

## Results

### Yield

In 2015–16, the average actual yield of Boro rice was the highest in Thakurgaon and the lowest in Sherpur (Table 3). The majority of the fields in Thakurgaon were under BRRI dhan29, which has high attainable yield [52] with long growth duration (approx. 165 days). Tanore, where Jirashail variety was grown, had similar average yield like Minikit in Sherpur. The Hybrid varieties gave the highest (7.39 t/ha) yield, followed by BRRI dhan29 (6.72 t/ha), BRRI dhan28 (6.14 t/ha) and other varieties (Minikit, Jirashail and Kajallata) whose yield ranged from 4.87 t/ha to 5.09 t/ha in 2015–16 (Table 3). The average attainable yield of BRRI dhan28 and BRRI dhan29 is 6.0 t/ha and 7.5 t/ha, respectively [52]. Thus, the observed yield was fairly close to the attainable yield for BRRI dhan29 and slightly higher for BRRI dhan28. In 2016–17, the average yield of BRRI dhan29 (6.81 t/ha) was higher than that of Hybrid rice (6.05 t/ha). BRRI dhan28 produced a lower yield (5.33 t/ha) in 2016–17 than in 2015–16. Minikit had a slightly higher yield (5.18 t/ha) in 2016–17 than in 2015–16.

**Table 3:** Number of fields and mean actual or observed yield of Boro rice under different sites, pump-types, rice varieties and transplanting dates.

Item	2015–16		2016–17	
	No. of fields	Yield (t/ha)	No. of fields	Yield (t/ha)
**Sites**				
Ishurdi	50	6.55	39	6.99
Kaharol	24	5.93	–	–
Mithapukur	69	6.92	62	5.50
Sherpur	52	5.00	46	4.43
Tanore	22	5.04	–	–
Thakurgaon	18	7.24	18	6.70
Badarganj	–	–	20	6.29
**Rice variety**				
BRRI dhan28	57	6.14	48	5.33
BRRI dhan29	61	6.72	43	6.81
Hybrid	43	7.39	28	6.06
Jirashail	22	5.04	–	–
Kajallata	21	4.87	–	–
Minikit	31	5.09	66	5.18
**Pump type**				
STW-Electric motor	80	6.62	74	6.11
STW-Diesel engine	115	5.89	73	5.00
DTW-electric	40	6.03	18	6.70
Solar power	–	–	20	6.29
**Transplanting date**				
January	107	5.87	60	5.35
February (1–14)	104	6.49	11	5.92
February (15–28)	24	6.05	12	6.18

Rice fields under electric motor-operated STWs produced the highest yield (6.62 t/ha) followed by electric motor-operated DTWs (6.03 t/ha) and diesel-operated STWs (5.89 t/ha). The mean and median yields across all rice fields were 6.16 t/ha and 6.13 t/ha, respectively in 2015–16 with a skewness coefficient of 0.83. In 2016–17, the mean and median yields were 5.75 t/ha, 5.93 t/ha with a skewness coefficient of -0.76. In the 2015–16, there was significant (p≤0.05) variation in the average yield of rice across the sites and rice varieties (Table 4). The irrigation pump-types and planting dates did not significantly impact the average yield (Table 4). However, in 2016–17, there was significant variation in actual yield across different pump types in addition to sites and rice varieties.

**Table 4:** Variance in actual yield (t/ha) of Boro rice across the sites, pump-types, rice varieties and transplanting dates.

	2015–16				2016–17			
Source	df	MSS	F-value	p-value	df	MSS	F-value	p-value
Site	5	33.4	78.6	0.000***	4	41.6	120.7	0.000***
Pump type	1	1.3	3.0	0.087	1	11.5	33.3	0.000***
Rice variety	3	9.8	23.1	0.000***	3	6.5	18.8	0.000***
Transplanting date	2	0.1	0.1	0.876	2	0.5	1.5	0.227

***: p-value < 0.001.

### Enterprise budgeting

#### Representation of factor inputs in the production cost

**Fig 2:**
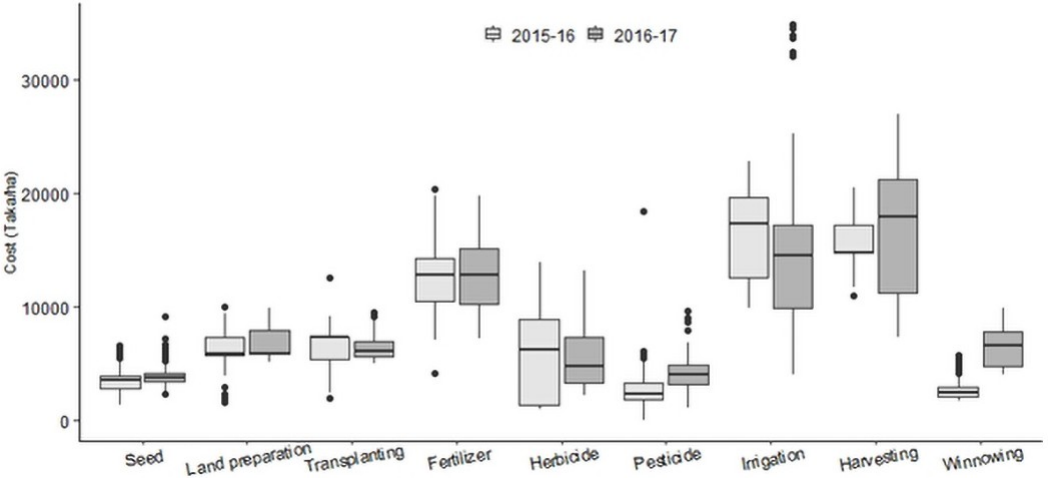
Variation of site-averaged total paid-out costs of different inputs: Seed (including seed, nursery preparation and seedling development), land preparation (tillage), transplanting (including seedling uprooting), fertilizer, herbicide (including weeding), pesticide, irrigation, harvesting (crop cutting, carrying, and threshing), winnowing (including drying) for Boro rice cultivation in 2015–16 and 2016–17. Boxes have horizontal bounds from first quartile (Q1) to third quartile (Q3). The horizontal line at the middle of the boxes represent the median. The dots represent the outliers in the data.

The input costs for rice cultivation varied across the monitored sites and between the two cropping years. Fig 2 illustrates the variation in total paid-out costs of different input categories per hectare for rice cultivation in 2015–16 and 2016–17 The variation was the highest for herbicide (CV = 62%) and pesticide (CV = 63%) in 2015–16 and for irrigation (CV = 56%) and herbicide (CV = 53%) in 2016–17. The lowest variation was for harvesting (including crop cutting, carrying, and threshing) in 2015–16 (CV = 11%) and for tillage and land preparation in 2016–17 (CV = 15%).

There was quite a high variation (11,873–20,810 Taka/ha in 2015–16 and 5,638–36,634 Taka/ha in 2016–17) in the average cost of irrigation across sites. This variation was due to the variation in pricing method of irrigation as given in Table 1. The highest variaton was always in Ishurdi and the lowest was in Thakurgaon. In Ishurdi, the pump owner charged 25% of total production of Boro rice as cost of irrigation for the whole cropping season. At the time of harvest, the pump owners harvest one-fourth of each field and take both rice and straw. Therefore, the cost of irrigation varies from field to field due to the variation in actual yield and straw. Due to higher (30% than 2015–16) price of rice in 2016–17, irrigation cost was also much higher than 2015–16. The main disadvantage of this pricing approach is that farmers pay more for irrigation if the yield and price of rice are higher. The advantage is that farmers do not need to invest in irrigation during the season. If both yield and price are low, then both the farmer and the pump owner bear the lower return, thus the cost of irrigation becomes low. Thakurgaon is a DTW site where farmers use a pre-paid card to irrigate their fields. So, the farmers have the liberty to irrigate as and when necessary. Due to high rainfall in 2016–17, the irrigation requirements was much lower [53] requiring less pumping of water to the rice fields. So the irrigation cost was much lower in 2016–17 than that of 2015–16. Rainfall during January to May (crop season) was 283 to 497 mm in 2015–16 and 304 to 527 mm in 2016–17 across the locations.

**Fig 3:**
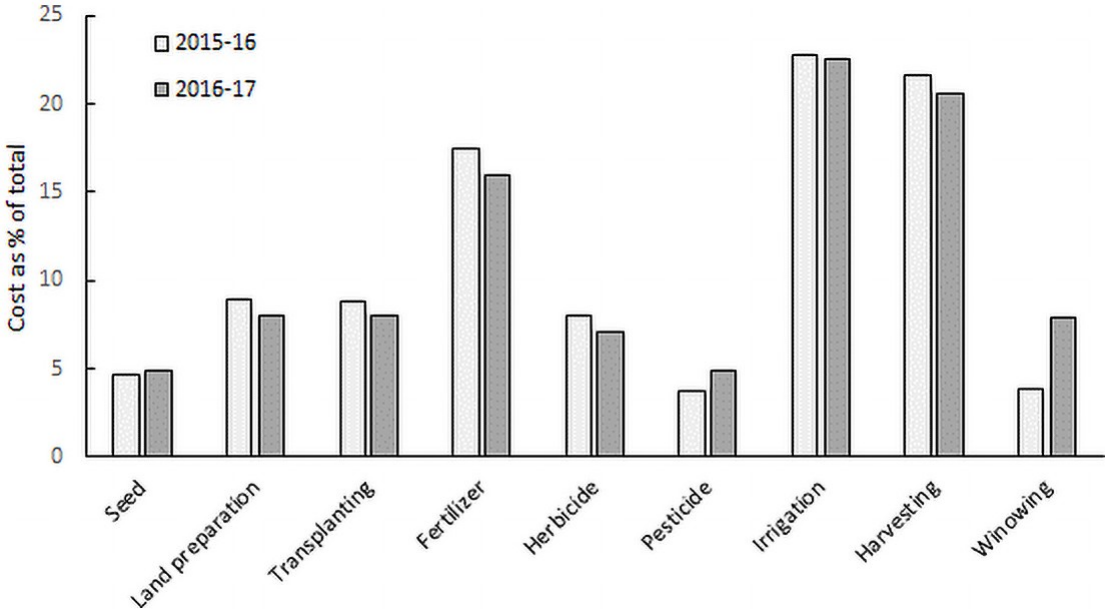
Paid-out costs of different inputs as percentage of total: Seed (including seed, nursery preparation and seedling development), land preparation (tillage), transplanting (including seedling uprooting), fertilizer, herbicide (including weeding), pesticide, irrigation, harvesting (crop cutting, carrying, and threshing), winnowing (including drying) for Boro rice cultivation in 2015–16 and 2016–17.

While there was variation in paid-out costs of different activities across the fields, their share as a percentage of total costs was similar each year (Fig 3). Total paid-out cost of production is highly dominated by the cost of fertilizer, irrigation, and harvesting (crop cutting, carrying, and threshing). These three activities accounted for 62% and 59% of the total cost respectively in 2015–16 and 2016–17. The cost of irrigation was the highest (23%) followed by harvesting (crop cutting, transporting, and threshing) (21–22%) and fertilizer (16–17%). Costs of cultivation were similar across varieties.

#### Gross benefit and income

**Fig 4:**
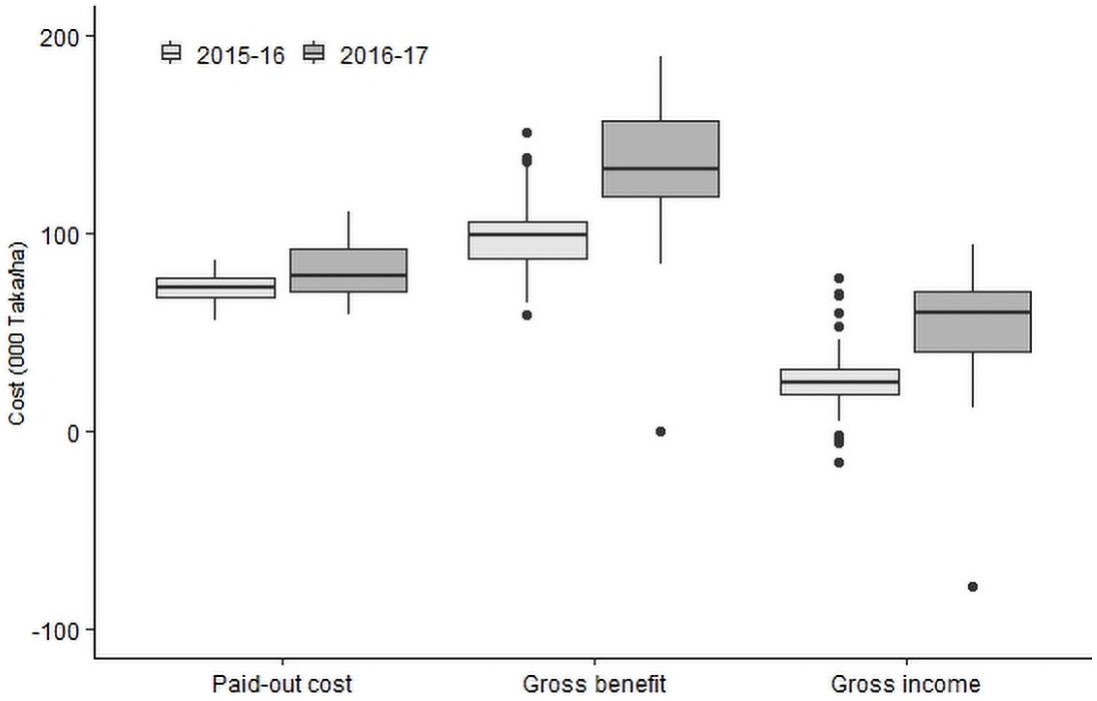
Variation of total paid-out cost, gross benefit, and gross income of Boro rice cultivation during 2015–16 and 2016–17 for all plots of all seven locations. Boxes have horizontal bounds from first quartile (Q1) to third quartile (Q3). The horizontal line at the middle of the boxes represent the median. The dots represent the outliers in the data.

**Fig 5:**
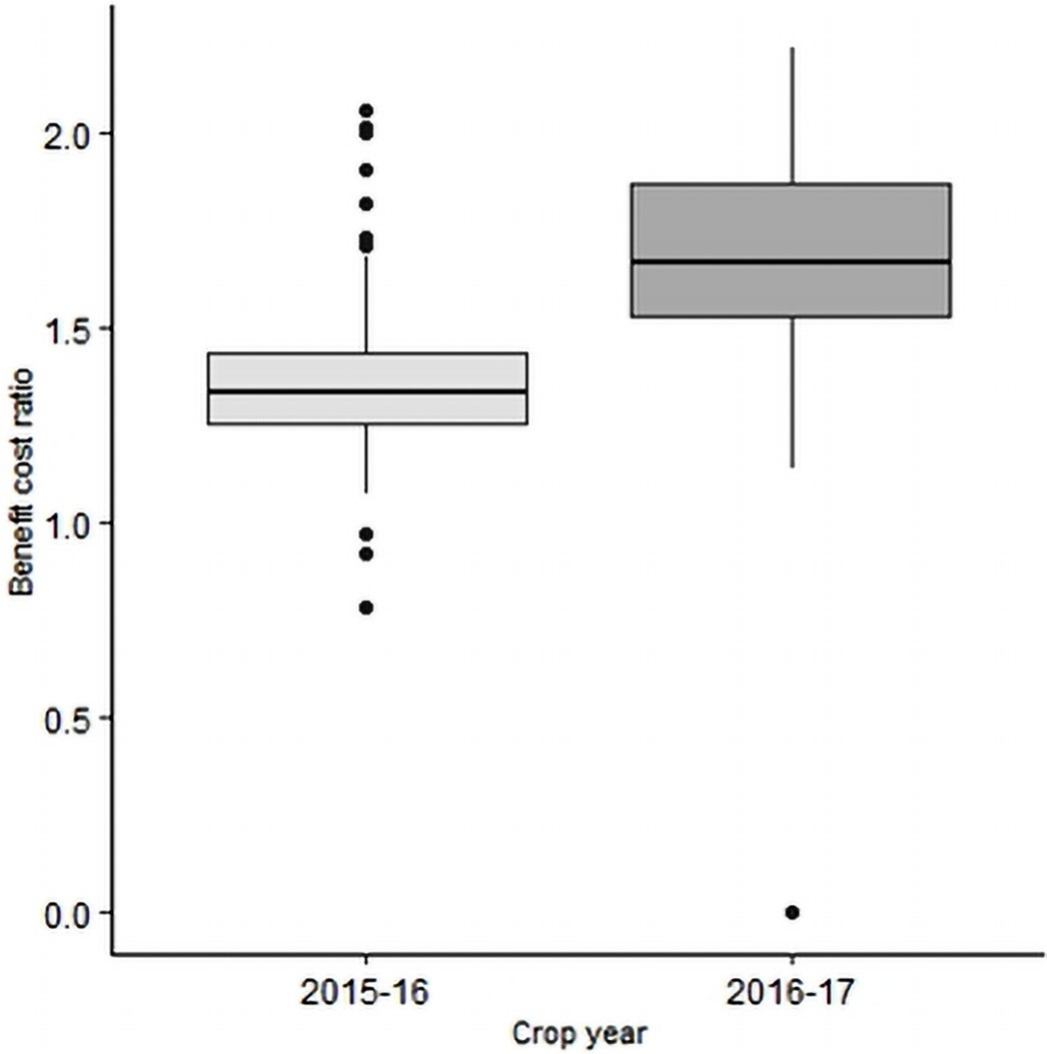
Variation in average benefit-cost-ratio of Boro rice cultivation during 2015–16 and 2016–17 for all plots of all locations. Boxes have horizontal bounds from first quartile (Q1) to third quartile (Q3). The horizontal line at the middle of the boxes represent the median. The dots represent the outliers in the data.

Total paid-out cost, gross benefit and gross income varied across the sites, irrigation pump-types, rice varieties and transplanting dates (Table 5). The variations in total paid-out cost, gross benefit and gross income of all rice fields are illustrated as box plots in Fig 4. Average gross income was the highest in Thakurgaon (30,893 Taka/ha in 2015–16 and 70,932 Taka/ha in 2016–17) in both years. The lowest gross income was in Kaharol (16,995 Taka/ha) in 2015–16 and Sherpur (29,927 Taka/ha) in 2016–17. The gross benefit was much higher (almost two-fold) in 2016–17 due to a 30% higher price of rice in that year. However, some fields did not recover the cost of production that is they had negative gross income as shown in Fig 4. There were few fields in 2015–16 which have a benefit-cost ratio less than one (Fig 5). In 2016–17, one field was completely damaged with no harvest.

**Table 5:** Cost and return of Boro rice across sites, rice varieties, pump-types, and transplanting dates.

Item	2015–16			2016–17		
	Total paid-out cost (Taka/ha)	Gross Benefit (Taka/ha)	Gross income (Taka/ha)	Total paid-out cost (Taka/ha)	Gross Benefit (Taka/ha)	Gross income (Taka/ha)
**Sites**						
Ishurdi	79,532	102,981	23,449	105,436	174,092	68,656
Kaharol	61,174	78,169	16,995	–	–	–
Mithapukur	70,134	96,982	26,848	78,821	134,762	55,941
Sherpur	69,588	93,387	23,799	79,646	109,574	29,927
Tanore	76,373	106,663	30,290	–	–	–
Thakurgaon	77,940	108,833	30,893	67,222	138,154	70,932
Badarganj	–	–	–	68,029	129,741	61,713
**Rice variety**						
BRRI dhan28	66,559	88,074	21,515	74,468	125,903	51,435
BRRI dhan29	78,231	103,949	25,717	84,429	151,708	67,278
Hybrid	72,582	100,344	27,762	78,836	143,397	64,561
Jirashail	76,373	106,663	30,290	–	–	–
Kajallata	71,880	91,076	19,196	–	–	–
Minikit	68,034	94,952	26,918	88,154	131,223	43,068
**Pump type**						
STW-Electric motor	70,975	107,640	25,317	90,558	152,923	62,365
STW-Diesel engine	71,519	96,291	23,000	81,662	121,492	39,830
DTW-electric	77,078	94,519	30,561	67,030	138,154	71,124
Solar power	–	–	–	68,029	129,741	61,713
**Transplanting date**						
January	72,780	97,219	24,439	86,416	131,612	45,196
February (1–14)	73,683	100,579	26,896	80,831	138,965	58,134
February (15–28)	63,971	83,995	20,025	70,624	139,218	68,593

The total paid-out cost varied significantly (p≤0.05) between the sites, pump-types, rice varieties, and transplanting dates in 2015–16 (Table 6). In 2016–17, this cost differed significantly only across the sites and pump-types. The gross benefit varied significantly across the sites and rice varieties in 2015–16, and across the sites, rice varieties and pump-types in 2016–17. The gross income also varied significantly across the sites, pump-types and rice varieties in 2015–16, and across the sites and rice varieties in 2016–17.

**Table 6:** Probability levels in the variance of total paid-out cost, gross benefit and gross income among the sites, pump-types, rice varieties, and transplanting dates.

Source	2015–16			2016–17		
	p-value for			p-value for		
	Paid-out cost	Gross benefit	Gross income	Paid-out cost	Gross benefit	Gross income
Site	0.000	0.000	0.000	0.000	0.000	0.000
Pump-type	0.007	0.007	0.057	0.000	0.709	0.000
Rice variety	0.000	0.012	0.035	0.051	0.001	0.000
Transplanting date	0.000	0.600	0.703	0.419	0.598	0.375

### Risk analysis

#### Stability in yield

**Fig 6:**
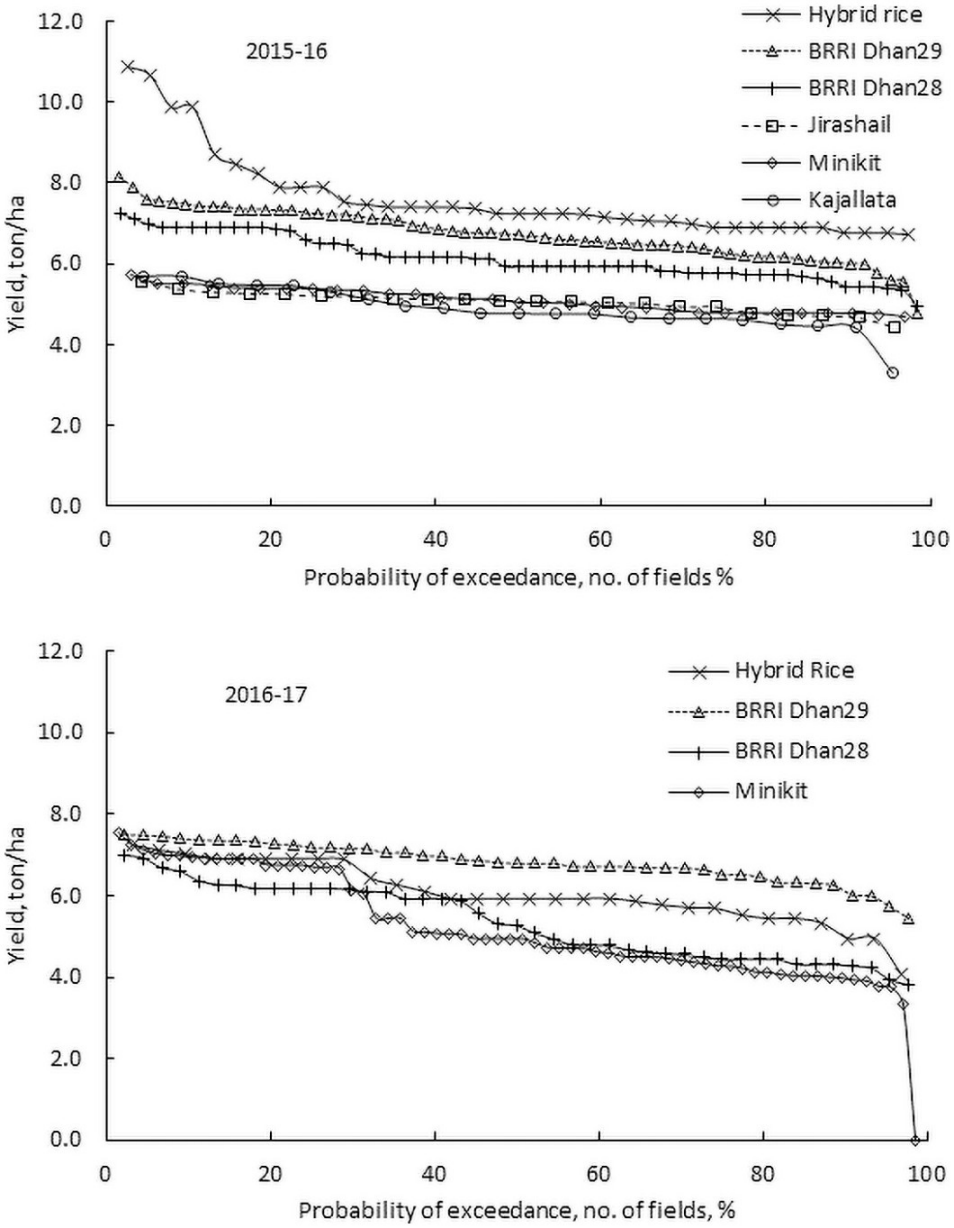
Probability of yields of different Boro rice varieties cultivated at different sites in 2015–16 and 2016–17.

There were significant differences among the average actual yield of Hybrid rice, BRRI dhan29 and BRRI dhan28 for 2015–16 (Tables 3 and 4) due to variation in potential performance across the varieties. Fig 6 shows the probability of exceedance of the observed yield of different varieties for all fields. The Hybrid rice is expected to result in 15–20% higher yields than the high yielding varieties [54–57]. The attainable yield of Hybrid rice is about 10 t/ha [54, 55]. However, in 2015–16, the probability of achieving the yield of 8.0 t/ha was only 20%. No field achieved 8.0 t/ha in 2016–17. Contrary to the previous year’s results, in 2016–17, the actual yield of Hybrid rice was significantly lower than BRRI dhan29 (Fig 6). The slope of the probability plot clearly indicates that the actual yield of BRRI dhan29 is much more stable than the other rice varieties across the locations and over the seasons. On the contrary, the yield of Hybrid cultivars was lower with higher variability in 2016–17. Though Hybrid rice has higher attainable yield than that of BRRI dhan29, its performance varied over the years. So, Hybrid rice is higher risk-prone than BRRI dhan29.

#### Stability in profitability

**Fig 7:**
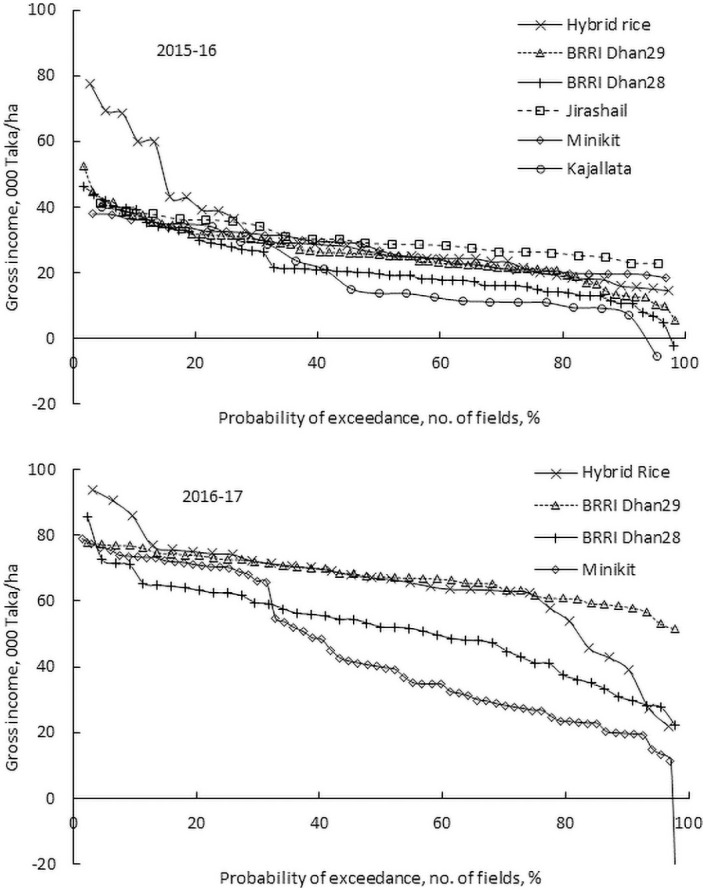
Probability of gross income of different Boro rice varieties cultivated at different sites in 2015–16 and 2016–17.

The probability distribution of gross income was different from the probability distribution of yield. In 2015–16, the average gross income was the highest for Jirashail. (Fig 7, Table 5). The average gross incomes of Kajallata and BRRI dhan28 were the lowest across the varieties cultivated. Hybrid rice, BRRI dhan29 and Minikit achieved similar average gross income. However, in 2016–17, the results were different. Among the 4 varieties of rice grown in that year, the average gross income was significantly lower for BRRI dhan28 and Minikit than BRRI dhan29 and Hybrid. There was lower variability in gross income in 2016–17 than in 2015–16 for both Hybrid rice and BRRI dhan29. The CVs of Hybrid rice and BRRI dhan29 were 27.1% and 10%, respectively in 2016–17. In 2015–16, the CVs were 47.6% and 34.7% respectively for BRRI dhan29 and Hybrid rice. The CVs of gross income for BRRI dhan28 and Minikit were 25.2% and 27.7% in 2016–17 than 51.6% and 23.6%, respectively in 2015–16. These results indicate that similar to yield, gross income of BRRI dhan29 is also much more stable than the other Boro varieties grown in the region. The performance (both yield and gross income) of Hybrid rice varied from year to year. Similarly, Jirashail and Minikit performed better (gave more gross income) in one season and poorly performed in the following season (Fig 7).

Due to the good grain quality, good taste, and slow-digesting character of BRRI dhan28 and Minikit, farmers prefer to grow these varieties for their consumption [16]. They have medium growth duration, 15–20 days shorter than BRRI dhan29 and Hybrid rice, with reasonable high attainable yield. Furthermore, in areas where three or more crops are grown in a year, cultivation of BRRI dhan28 as semi-subsistence farming is a good option despite lower gross income from this rice as BRRI dhan29 does not fit in the cropping pattern of those areas. For commercial rice cultivation, it would be better for the farmers to grow other varieties (Hybrid rice, BRRI dhan29, Minikit and Jirashail) if they can be accommodated in the cropping pattern. The gross income from these varieties is also similar to Minikit. However, Minikit has high demand in the market, and is easier to sell [16]. The selection of a variety of rice for cultivation is complex and depends on several other factors such as availability of good quality seeds, eating quality, market demand and price, transplanting window, agro-ecological conditions, local practices, etc.

#### Risk accounting

**Fig 8:**
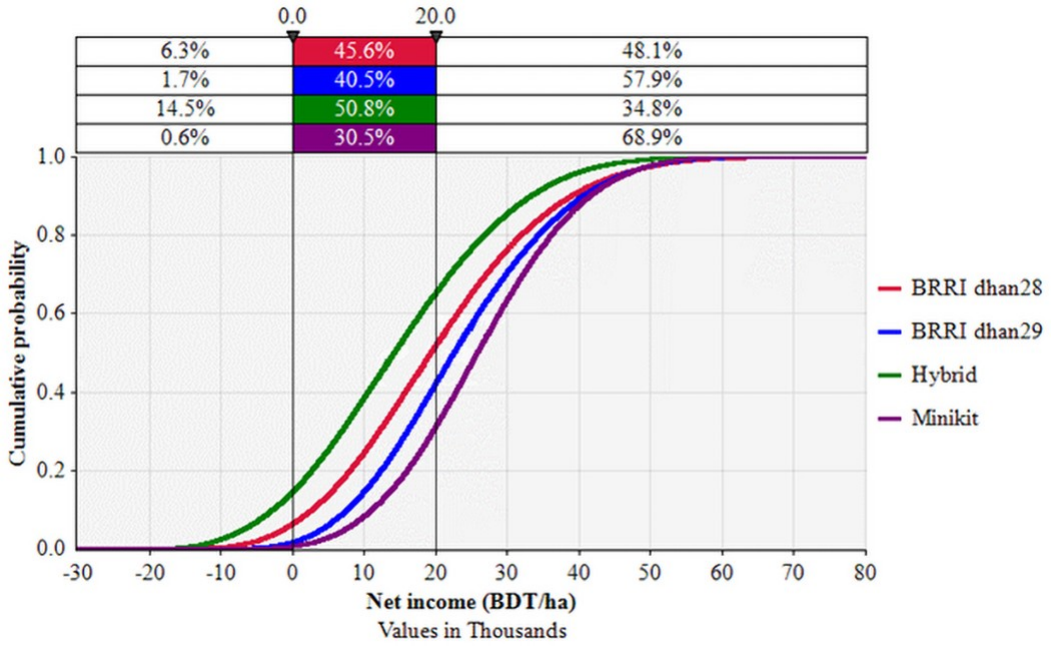
Cumulative probability distribution of net income of different Boro varieties during 2015–16 to 2016–17 in northwest Bangladesh.

For enterprise budgeting we estimated gross income for each cultivar. However, for stochastic budgeting, we estimated net income (gross income–total imputed cost) to evaluate relative risk and return status of popular Boro rice varieties. Table 7 shows the observed variability (2015–16 and 2016–17) in actual yield and price of BRRI dhan28, BRRI dhan29, Hybrid and Minikit estimated from the observation and were used in risk accounting. Fig 8 presents cumulative probability distribution function (CDF) of net income per hectare for major cultivars of Boro rice in NW Bangladesh. The CDF of Minikit shows first-degree stochastic dominance over the other cultivars (BRRI dhan28, BRRI dhan29 and the Hybrid). CDF of BRRI dhan29 shows second-degree stochastic dominance over BRRI dhan28 and Hybrid, indicating that Hybrid cultivar is the riskiest followed by BRRI dhan28 and BRRI dhan29. Farmers have over 15% chance of having negative net income from Hybrid cultivars. The probability of having negative net income for BRRI dhan28 decreased to 6% and further decreased to only 2% for BRRI dhan29. The results of risk analysis indicate that likelihood of having negative net income for Hybrid cultivars was the greatest followed by BRRI dhan28 and BRRI dhan29 under the market and environmental conditions during 2015–16 and 2016–17. However, the chance of having negative net income was almost zero for Minikit, which was economically more viable followed by BRRI dhan29 (Fig 8).

**Table 7:** Seasonal variability (2015–16 and 2016–17) in actual yield and price of BRRI dhan28, BRRI dhan29, Hybrid and Minikit.

Rice variety	Grain yield (t/ha)			Grain price (Taka/t)		
	Best	typical	Poor	High	typical	Low
BRRI dhan28	6.53	5.68	4.46	24,655	18,479	16,288
BRRI dhan29	7.57	6.79	5.52	22,887	17,867	15,143
Hybrid	8.00	6.78	6.35	20,750	15,959	12,150
Minikit	6.22	5.61	4.52	26,837	22,372	19,435

## Discussion

### Variation in yield

The study shows significant variability in actual yield of rice across fields, sites and varieties (Table 4). For the same variety, the difference in yield between fields at the same site is a result of crop management practices, diverse soil environments and seasonal weather conditions. FAO [58], Kabir et al. [59, 60] and Peng et al. [61] reported that biophysical factors, along with seasonal weather variation, cultural practices, socio-economic conditions, and institutional and policy issues are responsible for variation in rice yield. Farmers’ knowledge of the management of production factors is also crucial to achieving good yield [2]. The relative contribution of the major production factors, such as fertilizer, weed control, and pest (insect and disease), on the yield of Boro rice, was reported to be 0.52, 0.44 and 0.04, respectively [62]. The difference in major production factors is evident in the range of input costs across the fields (Fig 2). Other management factors such as suitable planting time, good quality seed, appropriate planting density, and balanced fertilizers are also important to maximize rice yield [63]. Rice yield was also related to the adequacy of irrigation application and operation mode of irrigation wells [53]. Of all the constraints on Boro rice cultivation, timely application of adequate irrigation water is the most important one, as rice is very sensitive to water deficit [64]. DTWs extract water from deeper aquifers using a submersible pump, and a drop in water level does not significantly impact pumping. DTWs are also operated by electric motors, which are more reliable than diesel engines and the price of water has also been shown to be cheaper for DTW’s [16]. As a result, there is usually adequate water for timely irrigation of fields using DTW’s. Mainuddin et al. [53] compared crop water requirement with irrigation supply of the same fields and found that DTW irrigated fields were generally oversupplied while STW fields were undersupplied in some locations. STW pumps have a smaller discharge capacity (Table 1) and use suction or centrifugal pumps. During the peak water demand month of March and April, groundwater level sometimes falls below the suction limit [16, 65, 66] making it difficult for the farmers to pump water. Irrigation application is often delayed, that affects rice yield. Bell et al. [23] showed that access to irrigation water has the greatest impact on Boro yield. Neumann et al. [25] also mentioned that differences in grain production efficiencies are significantly correlated with irrigation, along with accessibility, market influence, and agricultural labor.

The duration of transplanting of Boro rice in 2015–16 was longer; late December until end of February. In 2016–17 the transplanting period was shorter; 14 January to 18 February. Date of transplanting affected the yield of rice [53]. In general, delay in transplanting resulted in reduction in actual yield for BRRI dhan28 and BRRI dhan29, however not for Hybrid and other rice varieties of these fields [53]. BRRI [67] reported a decline in yield of BRRI dhan28 and BRRI dhan29 due to delay from the recommended transplanting period respectively of 25 December to 10 January and 15 December to 15 January. Biswas et al. [68] also reported that rice growth duration and yield decrease with a delay in planting date. The delay in transplanting exposes the flowering stage of rice to extremely high temperature, which reduces the yield significantly [69, 70]. So, the variation in actual yield between fields of the same variety can be attributed to some extent to variation in transplanting date, although the ANOVA test did not show a significant difference (Table 4). Some farmers delay transplanting, and transplant older seedlings due to unavailability of land and also to reduce the production cost, particularly irrigation cost, by shortening growth duration [16].

Many fields achieved close to or slightly above the average attainable yields [52] for BRRI dhan28 (6.0 t/ha) and BRRI dhan29 (7.5 t/ha). This indicates that it is possible to achieve the indicated [52] attainable yield with adequate management and reduce the variation observed (Fig 6). Reducing variation in yield across the fields, varieties, locations, and irrigation well-types help increase production, profitability and sustainability and improve land- and labour-use efficiency. Improved production-management practices and extension services help address the climate change impacts and variability. Appropriate institutional interventions by the government are also vital [2]. Detailed field level information, such as provided in this study, is vital to guide appropriate policy interventions by Government.

### Variations in profitability

The input cost of production activities shown in Fig 2, in general, is the sum of the cost of factor inputs and the labour required for the application or implementation of the inputs. Some activities are impacted mainly by the cost of factor inputs (such as seed, fertilizer, and pesticides costs) and others, the cost of labour (such as transplanting, harvesting, carrying, and threshing). Among the rice production activities, tillage (98%), irrigation (>90%), pesticide application (>90%) and threshing (80%) have been largely mechanized [71], and hence they require less labour. The most labour intensive parts of rice production comprise transplanting [31], and harvesting (crop cutting, carrying, and threshing), where the combined cost is about 40% of the total labour cost of production [37]. In this study, transplanting and harvesting costs were 31% of the total paid-out cost of production in 2015–16 and 29% in 2016–17. The availability of agricultural labour is decreasing at 0.25–0.40% per annum in Bangladesh [72]. Agricultural labourers have decreased from 70% in 1991 to 40% in 2018 [12] and the wage rates have increased from 180 Taka per day in 2010 to 397 Taka per day in 2018 [17, 73]. However, during the peak time of transplanting and harvesting, there is an acute shortage of labour [74, 75] which pushes the labour price to much higher than the usual price which inflates the cost of production and even reduces yields as farmers are forced to delay transplanting. In 2019, due to low price of rice and higher cost of labour, many farmers found that it is not cost-effective to harvest rice as the cost of harvest could be more than the price of the harvested rice [76, 77]. To deal with the situation, the government inflated the purchase price of rice and instructed agencies to purchase directly from farmers and avoid the margin charged by the middleman. The government provides subsidies to reduce the input price of fertilizers, diesel, and electricity for irrigation [6, 78]. So, it is difficult to reduce the cost of production by reducing the cost of input materials but is possible by mechanizing the transplanting and harvesting operations. Islam [79] showed that 40 man-days of labour could be saved for each hectare of rice cultivation by introducing transplanter and harvester machines, thereby reducing the cost of Boro rice cultivation substantially [31]. The use of mechanical harvesters can also reduce grain losses of about 5% per season [80]. Strengthening of farm mechanization can play a pivotal role in increasing rice yield and profitability [31, 81]. In this regard, public intervention, through providing subsidies for farm mechanization, and private intervention through developing entrepreneurship, is a prerequisite [81].

The variation in paid-out cost, gross benefit, gross income, and benefit-cost ratio reflects variations in input costs, yield and selling price of rice. The gross income and benefit-cost-ratio were significantly higher in 2016–17 than those in 2015–16. BRRI [82, 83] also reported that returns of Boro rice cultivation were notably higher in 2016–17 than 2015–16. Despite large and variable input costs, rice production is still profitable in the NW region of Bangladesh, as was also reported for the central area [27] and the southwest region [59]. The increasing cost of irrigation water, labour and other inputs have negatively impacted the production of Boro rice in recent years. Besides, spatial variation in productivity, fluctuating price of rice [6], and variation in profitability have added uncertainty to the sustainability of Boro rice cultivation.

### Prospect of rice cultivation

Rice is the most important crop of Bangladesh [10]. The production and price of rice are intricately linked with the political stability of Bangladesh [38]. This was proven during the 2008 global increase in the price of rice, when the then Government of Bangladesh found it hard to source imports from the global market [29, 40], fueling agitation by the people [84]. In 2017, 160,170 ha of Boro rice was damaged by floods in the northeast Haor region, that immediately caused an increase in market prices [85]. This is one of the main reasons for the increase rice price in 2016–17. The government reduced rice import taxes to stabilize the market (https://www.globaltimes.cn/content/1061703.shtml). In 2019, the market price of rice fell, and harvesting was uneconomical since the cost of harvesting, threshing, and winnowing exceeded income [76, 77].

The population of Bangladesh will increase to 202 million by 2050 [8, 18] and about 46 million tons of rice will be required to feed the population [7]. It is expected that a major portion of this will come from the irrigated Boro rice [7]. Sustaining production of Boro rice is of utmost importance. Current production in Bangladesh is not sufficient to meet future food demand, due to a combination of reduced cropped area and increased demand [8]. It is therefore important that yield and profitability are improved. To increase the yield of Boro rice, the government and the private sector are promoting the cultivation of Hybrid rice [34, 40] due to its higher yield [54–57]. The probability analysis of yield and gross income of this study showed that yield of Hybrid rice was highly variable and the gross income was lower than the most other varieties in 2015–16 (Figs 6 and 7). In 2016–17, the average yield and gross income were lower than BRRI dhan29. Farmgate prices for hybrid rice have in the past fallen 10%–20% below the price for other coarse rice varieties [40], although in this study the farmgate price was considered the same as that of BRRI dhan29 and BRRI dhan28. Stochastic risk accounting, using the data of two seasons, also shows that Hybrid rice is the riskiest variety. It is unlikely that farmers will benefit economically by cultivating Hybrid rice. Bangladesh Rice Research Institute (BRRI) has developed new varieties of Boro rice, the yield of which is higher than BRRI dhan29 [86]. The new variety, BRRI dhan92, with the similar characteristics to BRRI dhan29, has average attainable yield of 8.4 t/ha, which could rise to 9.3 t/ha under optimal management (http://knowledgebank-brri.org/wp-content/uploads/2014/02/BRRI-dhan92.pdf). Farmers will benefit by replacing current varieties with the new variety. In addition to increasing yield and reducing the cost of production, rice profitability can be improved by stabilizing the price of rice, which is volatile in Bangladesh [6] as there are almost equal numbers of sellers and buyers. The rice marketing chains in Bangladesh are generally long and complex owing to the participation of many small-scale stakeholders [11]. Private traders operating at different levels can manipulate the market leading to increased and unstable rice prices. Poor Bangladeshi households, both buyers and sellers of rice, feel the economic consequences of such manipulation [11].

The sustainability of Boro rice cultivation in the North-West region is expected to be significantly affected by the availability of irrigation water, the impact of climate change, and the availability of agricultural land. Groundwater is the dominant irrigation source (97% of the total area) for Boro rice in the North-West region [13, 16]. There is evidence that groundwater levels are declining in some parts of the region questioning the sustainability of groundwater irrigation in the future [65, 66, 87, 88]. The predicted increase in potential crop evapotranspiration and irrigation requirements, due to climate change [69, 89], and the declining trend of rainfall [88] are expected to exacerbate the situation further unless sustainable ways of groundwater irrigation are found and implemented. The yield of Boro rice is also predicted to be affected by the impact of climate change in the future [90–93]. Probably the biggest risk [7] to the sustainability of rice cultivation is the conversion (about 1% per year) of productive agricultural land to other uses [94, 95]. All these factors were, however, outside the scope of this study.

## Conclusions

Irrigated Boro rice production in the North-West region of Bangladesh is crucial for the country’s self-sufficiency in rice production. Boro rice yield, paid-out cost, gross benefit, and gross income varies in between rice varieties, transplanting dates, farmland sites and irrigation well-operating modes. We monitored actual Boro rice production factors in 420 fields across the region over two consecutive crop seasons and analyzed the yield, profitability, and risk. Although other studies have addressed the issues of Boro rice production, very few of them did risk analysis of varieties and have used such comprehensive data collected directly from farmland which stands our findings on a stronger footing. Our results show that uncertainty of input costs, productivity and profitability are serious concerns for sustaining Boro production. Amongst the input costs, irrigation is the highest contributor to the production cost (20–25%). This implies that government policies must target affordable irrigation water availability to the farmers. We also observed that the costs of inputs do not vary in between cultivating different rice varieties but are increasing in recent years which needs to be stabilized to minimize farmers’ risks. The trend of increasing labor cost for transplanting and harvesting, which totals about 40% of the labor cost of production, is likely to continue in the future. Efforts must be made to mechanize the transplanting and harvesting operations.

We found that Hybrid rice and BRRI dhan29 have the highest yields amongst different cultivated varieties. However, Hybrid rice yield was found to be most unstable, and farmers gross income was lower than the most other varieties. Thus, the government and the private sector’s push to promote the cultivation of Hybrid rice needs to be carefully monitored and re-evaluated. Interestingly, we found that BRRI dhan29’s yield and gross income are comparatively stable across the sites and over the years. However, this variety may only be suitable for commercial farming since it has a longer growing period and may not fit in farmers cropping calendar in places where three or more crops are grown in a year. Another option for commercial farming is Minikit cultivation which has zero negative net income. For semi-subsistence farming, BRRI dhan28 is a good option. Another major finding of the research is that the variation in the market price of rice has a serious impact on overall profitability. Farmers gross benefit increased twofold in 2016–17 than the previous year primarily due to the higher market price. This result highlights that the market price needs to be adequate and stable to ensure farmers profitability and sustainability.

In short, we recommend adoption of context-specific interventions to ensure affordable irrigation access, mechanization of transplanting and harvesting operations, choice of appropriate rice variety and stable input and market price to keep rice production profitable in northwest Bangladesh. The results and recommendations, though based on data collected from North-West Bangladesh, are relevant for entire Bangladesh and expected to help guide policymakers, advisors, and farmers.
